# Towards Novel Nutritional Strategies in Gestational Diabetes: Eating Behaviour and Obesity in Women with Gestational Diabetes Compared with Non-Pregnant Adults

**DOI:** 10.3390/nu15194141

**Published:** 2023-09-25

**Authors:** Laura C. Kusinski, Patrycja Tobolska, Danielle L. Jones, Nooria Atta, Elizabeth H. Turner, Hannah B. Lewis, Linda M. Oude Griep, Fiona M. Gribble, Claire L. Meek

**Affiliations:** 1 Wellcome-Trust MRC Institute of Metabolic Science, University of Cambridge, Cambridge CB2 0QQ, UK; lck34@cam.ac.uk (L.C.K.); patrycjatobolska@hotmail.com (P.T.); dlj36@medschl.cam.ac.uk (D.L.J.); na584@cam.ac.uk (N.A.); eht27@medschl.cam.ac.uk (E.H.T.);; 2 MRC Epidemiology Unit, University of Cambridge, Cambridge CB2 0QQ, UK; 3 Cambridge Universities NHS Foundation Trust, Cambridge CB2 0QQ, UK

**Keywords:** gestational diabetes, emotional eating, eating behaviour, maternal obesity, postpartum, diet, nutrition, glucose

## Abstract

Background: Gestational diabetes is associated with increased risk of obesity, type 2 diabetes and cardiovascular disease. Effective nutritional strategies are needed to reduce BMI and improve long-term maternal cardiometabolic health, but the relative contribution of maternal eating behaviour, a potential barrier to dietary change, has not been explored. We compared eating behaviour in women with gestational diabetes with that of men and non-pregnant women with comparable risk factors, and tested associations between eating behaviour traits and BMI in women with gestational diabetes. We hypothesized that eating behaviour would be unfavourable in gestational diabetes and would be associated with BMI. Methods: Participants (*n* = 417) including 53 men, 164 non-pregnant women and 200 women with gestational diabetes (singleton pregnancy; 29 weeks’ gestation) were recruited into three prospective studies assessing weight loss interventions, with similar entry criteria. The three-factor eating questionnaire (TFEQ-R18) assessed uncontrolled eating, emotional eating and cognitive restraint at study enrolment. Associations between BMI at study enrolment and TFEQ-R18 (% maximum score) were assessed using linear regression. Results: Women with gestational diabetes had significantly lower uncontrolled eating scores vs. men (53% vs. 65%; *p* < 0.001) and non-pregnant women (53% vs. 66%; *p* < 0.001), lower emotional eating scores vs. non-pregnant women (60% vs. 71%; *p* < 0.001) and higher cognitive restraint (*p* < 0.001 vs. men and non-pregnant women). In women with gestational diabetes, emotional eating scores were positively associated with BMI at study enrolment (beta coefficient 7.8 (95% CI 3.9 to 11.7), *p* < 0.001). Conclusions: Women with gestational diabetes have favourable eating behaviour compared with other population groups. Because BMI at study enrolment was associated with emotional eating, nutritional strategies which reduce emotional eating may provide new opportunities to improve long-term maternal health after gestational diabetes.

## 1. Introduction

Gestational diabetes, defined as hyperglycaemia with first onset or recognition in pregnancy, affects 20 million women worldwide per year and is strongly associated with dietary habits [1,2]. For example, increased risk of gestational diabetes has been associated with obesity and increased gestational weight gain [3] and pre-pregnancy dietary patterns characterised by high saturated fat intake or low carbohydrate intake [4,5,6]. Changes to maternal diet are a foundational aspect to gestational diabetes management internationally. However, despite the importance of diet in gestational diabetes, the role of eating behaviour in disease aetiology and management has not been explored. 

Barriers to studying eating behaviour include challenges in describing and quantifying behaviours and also in translating results into clinical practice. Over the last few decades, several constructs describing dietary behaviour have been used, such as cognitive restraint (CR), uncontrolled eating (UE) and emotional eating (EE), which can be quantified using the three-factor eating questionnaire (TFEQ-R18), validated for use in the general population [7], in obesity [8] and used widely in pregnancy [9,10]. These behaviour traits have been identified as directly contributing to obesity, independently of age, sex, socio-economic status and physical activity [11]. The TFEQ-R18 reflects eating behaviour at the time of questionnaire completion only. Eating behaviour is thought to be fairly consistent throughout adult life. Limited data exist about longitudinal changes in eating behaviour in pregnancy but suggest that most women have stable eating behaviour during pregnancy [12]. 

UE is a temporary loss of control over food intake, the inability to resist the desire to eat, or extreme feelings of hunger and eating in the absence of hunger. Common examples of UE include eating in response to boredom, habit, external stimuli or overeating [8,13]. EE describes the tendency to over-eat in response to negative emotions, and is related to UE, but has a distinct influence on BMI [14]. CR is the tendency to consciously control or restrict food intake and can be flexible or rigid. An individual with rigid restraint will tend to eliminate all fattening foods in an ‘all-or-nothing’ manner [13]. A flexible pattern of restraint will permit the consumption of fattening foods but in limited quantities, which shows a better association with long-term weight maintenance [13]. Although restraint and UE appear to be opposites, they can often occur in the same individual. In fact, individuals with a rigid pattern of restraint often have periods of UE. Flexible restraint however, where unhealthy foods are permitted but in limited quantities, is known to be a healthier approach to weight maintenance and can counteract the negative effects of UE [13,15]. The combination of high restraint with high UE has been recognised as a particular subtype of eating behaviour and may be associated with negative affect and EE [16,17]. However, restraint with low levels of UE is a key characteristic of people who lose weight and maintain the weight loss [18].

Relatively few studies have assessed eating behaviour in pregnancy, particularly in populations who might be considered at high risk of unfavourable eating behaviour. Furthermore, pregnancy commonly affects mood, emotional state and dietary choices, so might be particularly likely to influence eating behaviour. The TFEQ-R18 has been used in pregnancy studies to assess associations with gestational weight gain [9,19]. A small study has identified that behaviour traits such as UE may have associations with foetal growth and size at birth [20]. A better understanding of eating behaviour traits in women with gestational diabetes could help shape appropriate dietary management strategies for this group, during pregnancy and postpartum. 

The aim of the current study was to assess eating behaviour in women with gestational diabetes, in comparison with men and non-pregnant women with comparable risk factors, and to assess if eating behaviour was associated with BMI. We hypothesised that eating behaviour would be unfavourable in women with gestational diabetes compared with a cohort of overweight men and non-pregnant women and would be strongly associated with BMI. 

## 2. Materials and Methods

### 2.1. Study Design and Ethical Approvals

Participants were included in this analysis from enrolment data of three separate studies of weight loss interventions (studies compared in Table S1). We chose to compare women with gestational diabetes with men and non-pregnant women, representing the majority of other adults in the population. 

Pregnant women with gestational diabetes: women with gestational diabetes and a BMI ≥25 kg/m^2^ prior to 28 weeks of gestation were recruited to the DiGest study (*n* = 200), a multicentre randomised double-blind controlled trial of a reduced energy diet in pregnancy which has been described more fully elsewhere (ISRCTN 65152174) [21]. Women were recruited at antenatal diabetes clinics (in seven hospital sites in the east of England, including Addenbrooke’s Hospital), and were non-fasting at the time of study enrolment. Data at study enrolment were used for the current analysis, including measured BMI at study enrolment (mean 23 (SD 7.2) weeks’ gestation). The study is being conducted in accordance with the Declaration of Helsinki, the protocol was approved by the Research Ethics Committee (Reference 18/WM/0191), and all participants were provided with written information about the study and gave their written informed consent prior to participation. 

Male and non-pregnant female participants with normal glucose tolerance aged 18–65 years old (*n* = 57) were recruited using advertisements placed in Addenbrooke’s Hospital and the University of Cambridge to test a novel dietary intervention, encapsulated nutrients [22]. In order to fulfil the entry criteria, healthy volunteers were required to be free from other chronic diseases and recent acute conditions such as diarrhoea or constipation and have a BMI of ≥18.5 kg/m^2^. Participants were either taking no medication or were stable on medication which was considered unlikely to interfere with the results of the study. Participants with other forms of diabetes or endocrine disorders were excluded from this part of the study. The volunteers attended the hospital research centre after an overnight fast and undertook a standardised meal test with assessment of glycaemia. Data at study enrolment were used for the current analysis. The study was given ethical approval by the Research Ethics Committee (Reference 13/EE/0195) and all participants were provided with written information about the study and gave their written informed consent prior to participation.

Male and non-pregnant female participants who were overweight or obese (*n* = 191; BMI ≥ 25 kg/m^2^; age ≥ 18 years old) were recruited as part of a televised study which was designed to identify different contributing causes to obesity within the population and to assess if different dietary strategies were more successful in some specific patient groups compared with others. Participants were recruited in Glasgow, Manchester and London. The volunteers attended the mobile research centre after an overnight fast and undertook a standardised meal test with assessment of glycaemia. Data at study enrolment were used for the current analysis. The study was given ethical approval by the Research Ethics Committee (Reference 14/WS/0089) and all participants were provided with written information about the study and gave their written informed consent prior to participation.

Data collection on eating behaviour: eating behaviour traits were assessed at the first visit using the validated three factor eating questionnaire, TFEQ-R18 [7], which produces subscale scores for CR, EE and UE. Each raw subscale score was divided by the maximum possible score for that trait in order to produce a result between 0 and 1, with results expressed as a proportion of maximum possible score for each trait. A score of 1 would indicate that the participant had the maximum score possible for each trait. 

Definitions: 

Gestational diabetes was defined as glucose intolerance with first onset or recognition in pregnancy [1]. Participants with gestational diabetes recruited before 30 + 6 weeks’ gestation with diagnosis based upon one of the following methods:

- A standard clinical 75g OGTT in accordance with the guidelines of the National Institute of Health and Care Excellence (NICE) including glucose ≥5.6 mmol/L (101 mg/dL) fasting and/or glucose ≥7.8 mmol/L (140 mg/dL) at 2 h [23].
- In women with previous gestational diabetes or who were unable to tolerate an oral glucose tolerance test, regular glucometer readings above NICE criteria targets [24].
- An HbA1c ≥ 39 mmol/mol during the COVID-19 pandemic, in line with the interim recommendations of the Royal College of Obstetricians and Gynaecologists [25].

### 2.2. Statistical Analysis

The association between variables was tested using univariable and multivariable regression. The main variables included in the regression analysis are the eating behaviour domains (UE, EE and CR), and BMI. We considered that BMI could be both a cause and effect of altered eating behaviour (dependent or independent variable). We chose to present results using BMI as a dependent variable predominantly, but our analysis was not designed to assess causation. We provided results for both unadjusted regression and following adjustment for age and BMI at study enrolment (and gestational age at enrolment for women with gestational diabetes). 

To examine the association between eating behaviour domains in different population groups, we used unadjusted regression and adjusted for age and BMI. Assumptions underlying regression were tested and the residuals were normally distributed for each analysis. Residuals were assessed for normality using visual symmetry and a quantile–quantile plot. Analysis was performed using STATA^®^ (version SE 16.1; StataCorp, College Station, TX, USA). Missing data were not imputed. A value of *p* < 0.05 was considered to be the threshold for statistical significance. Although all variables were analysed as continuous variables where possible, results are presented graphically as categorical variables. 

## 3. Results

### 3.1. Participant Characteristics

A total of 53 men, 164 non-pregnant women and 200 women with gestational diabetes were included in the study with demographic data shown in Table 1. Non-pregnant women had a significantly higher BMI compared with men (*p* = 0.038) and pregnant women with gestational diabetes (*p* = 0.003). Men and non-pregnant women were significantly older than women with gestational diabetes (men *p* < 0.001; non-pregnant women; *p* < 0.001). In the whole cohort, BMI was significantly positively associated with UE (*p* < 0.001) and EE (*p* < 0.001) and negatively associated with CR (*p* < 0.023). Age was positively associated with UE (*p* = 0.035).

### 3.2. Eating Behaviour in Women with Gestational Diabetes Compared with Other Demographic Groups

Women with gestational diabetes had a significantly lower mean UE score compared with men and with non-pregnant women (Table 1 and Table 2).

The EE score was also significantly lower in women with gestational diabetes compared with the non-pregnant women but similar in men. The mean CR score was higher in women with gestational diabetes compared with other groups. 

### 3.3. Associations between Eating Behaviour and BMI in Women with Gestational Diabetes Compared with Other Demographic Groups 

In men and non-pregnant women, UE and EE were positively associated with BMI (*p* < 0.001; Table 3). There was no significant association observed between CR and BMI in men, or non-pregnant women (*p* > 0.05). In women with gestational diabetes, BMI was associated with EE (*p* < 0.001), but showed no association with UE and CR.

## 4. Discussion

### 4.1. Statement of Principal Findings

Although gestational diabetes has been attributed to a suboptimal maternal diet, this study demonstrates that affected women have favourable eating behaviour compared with non-pregnant adults seeking obesity treatment, with lower scores for UE and EE. EE was strongly associated with BMI in women with gestational diabetes. 

#### 4.1.1. Strengths and Weaknesses of Study

This study has several strengths. We assessed eating behaviour in a large sample of men, non-pregnant women and women with gestational diabetes, including participants with a broad range of age and BMI. Detailed information on glucose homeostasis was available for all participants and people with type 2 diabetes or prediabetes were excluded. Data on glucose homeostasis were gathered contemporaneously to data on BMI and eating behaviour, reducing the likelihood of misclassification bias according to diabetes or obesity status. 

The participants were recruited from three separate studies, from wide geographical areas with slight differences in entry criteria. While we aimed to include people with a comparable age and BMI to women with gestational diabetes, there were small but significant differences between groups according to mean age and BMI at study enrolment. Furthermore, all participants were recruited into studies of novel interventions designed to promote weight loss, which supported the inclusion of people across a wide range of BMI classes. However, we did not include a group of pregnant women without gestational diabetes which would have been helpful [9,19]. This additional study population would have allowed us to determine whether the findings shown here are due to pregnancy itself, or whether they are unique to women with gestational diabetes. 

Further assessment of eating behaviour in healthy pregnancy is warranted, as it is unclear how pregnancy might dynamically influence eating behaviour. Eating behaviour data were based upon completion of the TFEQ-R18 at a single time point and may not be an accurate reflection of eating behaviour when measured longitudinally, especially in pregnancy, when eating behaviour may be more dynamically affected by gestation or foetal growth [19]. The TFEQ-R18 questionnaire is a well validated tool to use for such studies; however, it is important to note that the questionnaire is open to the personal interpretation of the questions and may be affected by language barriers or colloquialisms. This subjectivity may introduce some bias due to differences in interpretation of the question or the scale. In addition to this there is a social desirability bias which may have been introduced. The women in the study with gestational diabetes had received dietary advice to support optimal pregnancy outcomes and may have felt motivated to answer differently than the non-pregnant adults who are seeking help with obesity for longer-term health reasons. 

Another limitation of this study is that educational status was not assessed in the participants. It is possible that the elevated CR and lower UE seen in the women with gestational diabetes may be a reflection of the higher educational status of this group but this was not examined. In addition, we did not look at the influence of hormones on eating behaviour. This would have been an interesting aspect to examine and may have explained some of the male and female differences observed but it was beyond the scope of the current study. However, levels of EE were similar in women with gestational diabetes to those of men. This suggests that reproductive hormones, per se, are not necessarily driving EE. Further work is needed to assess if this is a temporary change in EE in pregnant women in response to dietary advice and a diagnosis of gestational diabetes.

As gestational diabetes is a short-term condition during pregnancy, most women were recently diagnosed and had received recent advice on healthy eating and dietary manipulation. Ideally, eating behaviour would be assessed prior to women being informed of the diagnosis, and subsequently, to identify how eating behaviour changed in response to the education provided. However, there are many unanswered questions about longitudinal changes in maternal diet in pregnancy, and how this might be influenced by medical conditions, health information or contact with healthcare professionals. There are logistical challenges in collecting eating behaviour data prior to diagnosis of gestational diabetes. Larger, longer-term studies will be required to clarify the importance of eating behaviour to maternal and offspring outcomes in pregnant women. One possible interpretation of our data would be that the recent advice on diet and healthy lifestyle given to women after diagnosis of gestational diabetes has promoted improved restraint and reduced UE and EE. However, the association of EE with BMI suggests that strategies to address EE might be an important addition to advice given on diagnosis of gestational diabetes in pregnancy and postpartum.

#### 4.1.2. Meaning of the Study and Implications for Clinical Care

Pregnancy is considered a receptive time to promote positive changes, such as stopping smoking, improving diet and increasing exercise levels [26], in health behaviours among women. The increasing incidence of maternal overweight and obesity has increased awareness of the dangers of existing dogma (“eating for two”) and promoted increased awareness of healthy eating. However, very few studies have described eating behaviour in pregnant women, or how pregnancy itself might influence eating behaviours in lean, overweight or obese women. One report compared 50 pregnant women and 50 non-pregnant nulliparous women [27]. The authors identified that pregnant women reported a higher dietary energy intake and had lower levels of CR compared with non-pregnant women [27]. Our data suggest that women with recently diagnosed gestational diabetes (*n* = 200) have higher levels of CR and lower levels of UE and EE compared with non-pregnant women (*n* = 181). 

Dietary choices in pregnancy are important to support healthy levels of gestational weight gain, which may improve perinatal outcomes for mother and child. Very few studies have examined the influence of maternal eating behaviour upon pregnancy health and offspring outcomes. Tang and colleagues studied 190 women from <10–36 weeks gestation and identified that disinhibition was associated with gestational weight gain, but that the association was attenuated by adjustment for socioeconomic factors and BMI at study enrolment [10]. Van der Wijden and colleagues identified that most women had stable eating behaviour from 15 to 35 weeks gestation with no association with gestational weight gain in 161 healthy women [12]. Plante and colleagues also identified no association with eating behaviour and gestational weight gain in 53 pregnant women but noted some longitudinal changes in eating behaviour during pregnancy, with a reduction in CR in the last trimester [19]. Jaakkola et al. have identified that eating behaviour is associated with habitual diet [28]. UE was associated with higher intake of energy, sucrose and fibre. Both UE and EE were associated with BMI at study enrolment [28]. Mumford et al. identified that preconception-restrained eating behaviour (measured using the revised restraint scale) was associated with excessive gestational weight gain in normal, overweight, and obese women [29]. 

Eating behaviour has not been consistently studied in pregnant women with conditions such as gestational diabetes, where diet may contribute to disease aetiology and is crucial for optimal disease management. A key aim of this study was to identify if maternal eating behaviour was likely to be a barrier to adherence to medical nutritional therapy, the first-line treatment for women with gestational diabetes. We identified that women had lower scores for UE than other groups and lower EE when compared with non-pregnant women, suggesting that most women with gestational diabetes have sufficient control over their dietary choices and eating behaviour to successfully modify their diet and manage their condition. Although this study used data from enrolment into the DiGest trial, further opportunities to assess eating behaviour, dietary choices and gestational weight change in women with gestational diabetes will be available once the trial is completed.

BMI can be considered to be a product of longer-term maternal eating behaviour. In women with gestational diabetes, EE was the main behavioural contributor to BMI, while CR had no association with BMI. While it is difficult to speculate about pre-diagnosis or pre-pregnancy eating behaviour in women who subsequently develop gestational diabetes, it is possible that interventions targeting EE could help women adhere to medical nutrition therapy in pregnancy or support weight loss postnatally. Further assessment of the importance of EE and related interventions in pregnancy and postnatally is warranted, both in healthy populations and in women with specific nutritional needs. 

## 5. Conclusions

Women with gestational diabetes have a favourable eating behaviour compared with other population groups. Because BMI at study enrolment was associated with EE, nutritional strategies which reduce EE may provide new opportunities to improve long-term maternal health after gestational diabetes.

## Figures and Tables

**Table 1:** Characteristics of men, non-pregnant women, and women with gestational diabetes, included in the current study. Assessment of eating behaviour included UE, EE and CR. Data are presented as mean (SD) or *n* (%) as appropriate. NA: not applicable.

	Men *n* = 53	Non-Pregnant Women *n* = 164	Women with Gestational Diabetes *n* = 200	*p* 1 vs. 2	*p* 1 vs. 3	*p* 2 vs. 3
Demographic Details						
Age years	38.8 (13.3)	36.8 (12.4)	32.6 (4.8)	0.248	<0.001	<0.001
BMI at study enrolment kg/m^2^	33.9 (8.8)	36.1 (8.6)	34.2 (6.2)	0.060	0.770	0.014
European Ethnicity %	Not collected	Not collected	156/200 (78.0)			
Glucose Homeostasis						
Normal Glucose Tolerance %	53/53 (100.0%)	164 (100.0%)	NA			
Gestational Diabetes Diagnosis and Treatment						
Gestational age at diagnosis weeks	NA	NA	23.0 (7.2)			
Gestational age at study enrolment weeks			29.7 (2.4)			
HbA1c at diagnosis mmol/mol	NA	NA	39.5 (4.4)			
On Metformin	NA	NA	44 (22.0%)			
On Neutral protamine hagedorn insulin	NA	NA	55 (27.5%)			
On Insulin aspart	NA	NA	23 (11.5%)			
Eating Behaviour (Standardised Scores)						
UE	0.65 (0.15)	0.66 (0.14)	0.53 (0.13)	0.699	<0.001	<0.001
EE	0.58 (0.24)	0.71 (0.21)	0.60 (0.22)	<0.001	0.588	<0.001
CR	0.48 (0.12)	0.51 (0.11)	0.62 (0.15)	0.157	<0.001	<0.001

**Table 2:** Comparison of eating behaviour in women with gestational diabetes mellitus (GDM) versus other groups using linear regression, unadjusted or adjusted for age and BMI. Data are presented as beta coefficient (95% confidence interval) significance, standard error (SE) and R-squared (Rsq). Significant associations have been shown in bold font.

	Unadjusted Analysis			Adjusted for Age and BMI		
	Uncontrolled Eating (UE)	Emotional Eating (EE)	Cognitive Restraint (CR)	Uncontrolled Eating (UE)	Emotional Eating (EE)	Cognitive Restraint (CR)
GDM group vs. Men	**0.12** **(0.08 to 0.16) *p* < 0.001** **SE: 0.02** **Rsq: 18.8%**	−0.02 (−0.09 to 0.05) *p* = 0.588 SE: 0.03 Rsq: 6.0%	**−0.14** **(−0.18 to −0.10) *p* < 0.001** **SE: 0.02** **Rsq: 16.7%**	**0.08** **(0.03 to 0.14) *p* = 0.004** **SE: 0.03** **Rsq: 15.3%**	−0.07 (−0.15 to 0.02) *p* = 0.113 SE: 0.04 Rsq: 13.2%	**−0.14** **(−0.20 to −0.08) *p* < 0.001** **SE: 0.03** **Rsq: 14.6%**
GDM group vs. Non-pregnant Women	**0.13** **(0.11 to 0.16) *p* < 0.001** **SE: 0.01** **Rsq: 18.8%**	**0.11** **(0.07 to 0.16) *p* < 0.001** **SE: 0.02** **Rsq: 6.0%**	**−0.11** **(−0.13 to −0.08) *p* < 0.001** **SE: 0.01** **Rsq: 16.7%**	**0.10** **(0.06 to 0.13) *p* < 0.001** **SE: 0.02** **Rsq: 15.3%**	**0.09** **(0.04 to 0.15) *p* = 0.002** **SE: 0.03** **Rsq: 13.2%**	**−0.11** **(−0.15 to −0.07) *p* < 0.001** **SE: 0.02** **Rsq: 14.6%**

**Table 3:** Associations between BMI and eating behaviour traits in participants categorised according to demographic group (men, non-pregnant women and women with gestational diabetes). Data are presented as beta coefficient (95% confidence interval), significance, standard error (SE) and R-squared (Rsq) using linear regression unadjusted and adjusted for age (and gestational age in gestational diabetes group). Significant associations have been shown in bold font.

		*n*	Uncontrolled Eating (UE)	Emotional Eating (EE)	Cognitive Restraint (CR)
Unadjusted Linear Regression					
BMI	Men	53	**26.5 (11.6 to 41.3), *p* = 0.001** SE: 7.4; Rsq: 20.4%	**13.1 (3.5 to 22.7), *p* = 0.009** SE: 4.8; Rsq: 13.0%	−1.7 (−23.0 to 19.6), *p* = 0.874 SE: 10.6; Rsq; <0.01%
	Non-pregnant Women	164	**19.1 (9.9 to 28.3); *p* = 0.009** SE: 3.1; Rsq: 4.2%	**8.2 (2.1 to 14.4), *p* = 0.009** SE: 3.1; Rsq: 4.2%	−8.1 (−20.1 to 3.9), *p* = 0.184 SE: 6.1; Rsq: 1.1%
	Women with gestational diabetes	200	1.9 (−4.9 to 8.8), *p* = 0.575 SE: 3.5; Rsq: 0.2%	**7.8 (3.9 to 11.7), *p* < 0.001** SE: 2.0; Rsq: 7.0%	−1.9 (−8.0 to 4.2), *p* = 0.539 SE: 3.1; Rsq: 0.2%
Adjusted linear regression					
BMI	Men	67	**26.5 (8.5 to 44.5), *p* = 0.005** **SE: 8.7; Rsq: 53.7%**	**14.5 (2.2 to 26.7); *p* = 0.023** **SE: 5.1; Rsq: 39.4%**	4.6 (−18.8 to 28.1); *p* = 0.689 SE: 11.4; Rsq: 37.7%
	Non-pregnant Women	181	**32.0 (18.9 to 45.2); *p* < 0.001** **SE 6.6; Rsq: 30.1%**	**12.9 (1.5 to 24.31), *p* = 0.027** **SE: 5.7; Rsq: 12.8%**	**−18.0 (−36.0 to 0.15); *p* = 0.052** **SE: 7.4; Rsq: 10.9%**
	Women with gestational diabetes	200	1.6 (−5.2 to 8.5); *p* = 0.636 SE 3.5; Rsq: <0.1%	**7.7 (3.7 to 11.6); *p* < 0.001** **SE: 2.0; Rsq: 7.6%**	−1.5 (−7.8 to 4.8); *p* = 0.636 SE: 3.2; Rsq: 0.4%

## Data Availability

Data are available upon request from the corresponding author, subject to study steering group approval.

## Supplementary Materials

The following supporting information can be downloaded at: https://www.mdpi.com/article/10.3390/nu15194141/s1, Table S1. Description of the three studies which were included in this analysis. Data are presented as mean (SD) or *n*(%) as appropriate.
