# Supplementary material for: Towards Novel Nutritional Strategies in Gestational Diabetes: Eating Behaviour and Obesity in Women with Gestational Diabetes Compared with Non-Pregnant Adults
Source: Nutrients. 2023 Sep 25;15(19):4141. doi: 10.3390/nu15194141 (PMC10574012; doi:10.3390/nu15194141)
Supplement: Supplementary file 1 [file nutrients-15-04141-s001.zip › nutrients-2530368-supplementary.pdf]

nutrients

MDPI

Supplementary Materials

Table S1. Description of the three studies which were included in this analysis. Data are presented as mean (SD) or n(%) as appropriate.

	**Baseline Data for Encapsulated Nutrients Study**	**Baseline Data for Personalised Weight Loss Study**	**Baseline Data for Dietary Intervention in Gestational Diabetes Study**
Study size	*n* = 43	*n* = 174	*n* = 200
Rationale	Attendance for study of gut hormones and potential weight loss intervention using encapsulated nutrients.	Attendance for study of potential weight loss intervention using personalised behavioural / pharmacological intervention.	Attendance for study of potential weight loss intervention using a whole-diet approach with energy restriction.
Entry criteria—BMI kg/m²	≥18.5 kg/m²	≥25 kg/m²	≥25 kg/m²
Entry criteria—age years	≥18	≥18	≥18
Entry criteria—health status	Healthy people or people with type 2 diabetes. Severe psychiatric or physical comorbidities excluded. Previous bariatric surgery excluded.	Healthy people or people with type 2 diabetes. Severe psychiatric or physical comorbidities excluded. Previous bariatric surgery excluded.	Gestational diabetes diagnosed; severe psychiatric or physical comorbidities excluded. Previous bariatric surgery excluded.
Nature of baseline visit	Fasting	Fasting	Non-fasting
Nature of eating behaviour questionnaire	TFEQ-R18	TFEQ-R18	TFEQ-R18
Time period	2014	2014–2016	2019–2022
Recruitment	Single centre study; local recruitment through advertising at hospital/University sites; Cambridge, UK	Multi-centre study; recruitment from advertising in Glasgow, Manchester and London, UK	Multi-centre study; recruitment from outpatient clinics in seven public hospital sites in the East of England, including Cambridge, UK
**Baseline Characteristics**	*n* = 57	*n* = 191	*n* = 200
% Female	31/57 (54.4)	150/191 (78.5)	200/200 (100.0)
BMI kg/m²	25.8 (4.9)	38.9 (7.1)	34.2 (6.2) at study entry.
Age years	36.3 (14.0)	41.8 (12.5)	32.6 (4.8)
Pregnant at study enrolment	0 (0.0)	0 (0.0)	200/200 (100.0)
Gestational age at diagnosis weeks	NA	NA	23.0 (7.2)
Gestational age at study enrolment weeks	NA	NA	29.7 (2.4)
Normal glucose tolerance	43/57 (75.4)	174/191 (91.1)	0 (0.0)
Pre-diabetes	6/57 (10.5)	11/191 (5.8)	0 (0.0)
Type 2 diabetes	8/57 (14.0)	6/191 (3.1)	0 (0.0)
Gestational diabetes	0 (0.0)	0 (0.0)	200/200 (100.0)
